# Reflexões históricas sobre varíola, ciência e saúde: entrevista com Tania Fernandes

**DOI:** 10.1590/S0104-59702026000100023

**Published:** 2026-08-07

**Authors:** Tania Maria Dias Fernandes, Tânia Salgado Pimenta, Gutiele Gonçalves dos Santos, Jaqueline Hasan Brizola

**Affiliations:** i Pesquisadora aposentada, Casa de Oswaldo Cruz/Fiocruz. Rio de Janeiro – RJ – Brasil. fernandestaniam@gmail.com; ii Pesquisadora, Casa de Oswaldo Cruz/Fiocruz. Rio de Janeiro – RJ – Brasil. tania.pimenta@fiocruz.br; iii Doutoranda, Casa de Oswaldo Cruz/Fiocruz. Rio de Janeiro – RJ – Brasil. gutielegoncalves12@gmail.com; iv Pesquisadora de pós-doutorado, Casa de Oswaldo Cruz/Fiocruz. Rio de Janeiro – RJ – Brasil. brizajaque@gmail.com

**Keywords:** Vacina, Varíola, Saúde pública, Vaccine, Smallpox, Public health

## Abstract

A entrevista com a professora Tania Maria Dias Fernandes, pesquisadora aposentada da Casa de Oswaldo Cruz/Fiocruz, explora sua trajetória acadêmica, que abrange estudos sobre a história das ciências, saúde pública e vacinas. Entre os temas discorridos pela entrevistada, destacam-se pesquisas sobre a institucionalização da vacina antivariólica, a erradicação da varíola, o papel da vigilância sanitária e os desafios históricos e contemporâneos enfrentados por comunidades marginalizadas. A entrevista também aborda as estratégias adotadas no processo de vacinação, os debates científicos que influenciaram as políticas de saúde e a importância de entender a saúde pública por meio de uma abordagem interdisciplinar.

## Abstract (en)

This interview with retired Casa de Oswaldo Cruz/Fiocruz researcher Tania Maria Dias Fernandes explores her academic trajectory, which covers studies on the history of science, public health and vaccines. Notable topics include the institutionalization of the smallpox vaccine, the eradication of smallpox, the role of health surveillance and the historical and contemporary challenges faced by marginalized communities. The interview also addresses strategies adopted during the vaccination process, scientific debates that influenced health policy, and the importance of understanding public health through an interdisciplinary approach.

## Contextualização da trajetória da entrevistada

A professora Tania Maria Dias Fernandes, pesquisadora aposentada da Casa de Oswaldo Cruz (COC), unidade técnico-científica da Fundação Oswaldo Cruz (Fiocruz), construiu trajetória acadêmica marcada pela interseção entre as áreas da saúde e das ciências humanas, demonstrando profundo comprometimento com a pesquisa histórica e a saúde pública no Brasil. Formou-se em farmácia pela Universidade Federal do Rio de Janeiro (UFRJ) em 1976 e concluiu seu mestrado na Escola Nacional de Saúde Pública (Ensp), em 1991. Seu interesse pela história social e pelo desenvolvimento científico a levou a realizar o doutorado na Universidade de São Paulo, concluído em 2001. Ao longo de sua trajetória na COC, onde atuou como pesquisadora, Tania Fernandes dedicou-se a estudar a história das ciências e das instituições científicas, com enfoque especial na história da vacina antivariólica e da erradicação da varíola. Suas contribuições acadêmicas abrangem a história da vigilância sanitária e das doenças no Brasil, bem como a história urbana e das favelas, destacando-se por sua pesquisa sobre as comunidades de Manguinhos. Desde 2005, a entrevistada investiga a história e a memória dessa região, publicando livros e artigos que colaboram para o entendimento das condições de vida em favelas e o impacto das políticas de saúde. Além disso, Tania coordenou um projeto sobre a constituição da saúde coletiva no Brasil, inicialmente em parceria com o Instituto de Saúde Coletiva da Universidade Federal da Bahia e, em uma segunda fase, com a equipe da COC/Fiocruz.

Como professora do Programa de Pós-graduação em História das Ciências e da Saúde, Tania Fernandes orientou importantes pesquisas e ministrou disciplinas sobre as temáticas aqui mencionadas. Em sua trajetória, destacamos o pós-doutorado realizado no Programa de Pós-graduação em Urbanismo, na Faculdade de Arquitetura e Urbanismo da UFRJ, no qual aprofundou seus estudos sobre o morar em favelas no Rio de Janeiro, consolidando sua *expertise* na análise urbana e de saúde. Em 2015, recebeu o Prêmio Abeu (da Associação Brasileira de Editoras Universitárias) pelo livro *Cidades saudáveis? Alguns olhares sobre o tema* (Silveira, Fernandes, Pellegrini, 2014), uma referência no campo da história da saúde e das comunidades urbanas no Brasil.

A entrevista com Tania Fernandes nos convida a refletir sobre a trajetória de uma historiadora da saúde, ao mesmo tempo que evidencia os caminhos da construção da historiografia da saúde na Fiocruz e no Brasil.

*Professora Tania, sua formação em farmácia, saúde pública e história reflete uma trajetória multidisciplinar. Como esses diferentes campos do conhecimento moldaram sua carreira acadêmica e suas pesquisas? O que a motivou a investigar esses temas e como eles se interconectam em seus estudos?*

Minha formação em farmácia, saúde pública e história reflete a multidisciplinaridade que envolve esses diferentes campos do conhecimento, que traduzi em pesquisas, por meio das quais direcionei meu trabalho e minhas reflexões na COC. Ao longo dos meus 37 anos de carreira na instituição, busquei temas de meu interesse ou que foram solicitados pela Casa, nos quais essa tríade disciplinar teve uma contribuição significativa, com enfoques diferenciados diante do amplo temário que se revelou com a consolidação da instituição. Considero que minha formação em microbiologia foi fundamental para o desenvolvimento de pesquisas sobre a história de doenças, como a tuberculose e a varíola, e também sobre a história do câncer, pois me permitiu compreender melhor textos científicos sobre esses temas. A formação em saúde pública me orientou a pensar políticas públicas, o movimento sanitário brasileiro, as instituições de saúde no país e a história da ciência, além de me permitir elaborar estudos sobre o espaço urbano e as favelas.

A abordagem histórica, à qual dediquei profunda atenção, representou uma guinada em relação à minha formação anterior em farmácia e saúde pública, abrindo novos horizontes, com questões e desafios desconhecidos, até então, para mim. Ainda na Ensp, nos primeiros passos na área de saúde pública, iniciei esse investimento em pesquisa sobre a história do câncer, que originou a publicação *História e saúde pública: a política de controle do câncer no Brasil* (Bodstein et al., 1987). Com a perspectiva de criação da COC, em 1986, a equipe que atuava nessa pesquisa foi convidada a construir a nova unidade, e então me inseri nesse projeto.

*Seu estudo sobre a história da vacinação antivariólica no Brasil foi pioneiro entre as pesquisas referentes a esse tema. Você poderia falar um pouco sobre o contexto em que começou a trabalhar com uma abordagem histórica? Você já era pesquisadora da Casa de Oswaldo Cruz?*

Meu ingresso nas reflexões com abordagem histórica ocorreu quando ainda estava na Ensp, antes da criação da COC, como já mencionei. Esse foi o projeto que abriu portas para minha entrada na COC, junto a outros colegas, quando fomos convidados a investigar, inicialmente, a história da Fiocruz. Buscamos documentos que ainda não haviam sido questionados pelo olhar de historiadores, e me deparei com uma série de ofícios e cartas que abordavam a vacina antivariólica e a relação do então Instituto Soroterápico Federal – posteriormente Instituto Oswaldo Cruz e, mais tarde, Fiocruz – com o Instituto Vacínico Municipal, este último responsável pela produção da vacina antivariólica no Rio de Janeiro (1887-1920). A partir dessa pesquisa, abriu-se para mim a possibilidade de estudar a vacina antivariólica, as relações institucionais e os debates travados entre os personagens envolvidos nos espaços científico, médico e político. Com o desenvolvimento de diferentes pesquisas, elaborei reflexões que abrangem desde a introdução da vacina no século XIX até a erradicação da doença em 1970, por meio de projetos independentes entre si.

A orientação institucional da Fiocruz e, em particular, da COC, ao incentivar a capacitação de seus profissionais, direcionou-me à conclusão do mestrado, cuja dissertação sobre a vacina antivariólica resultou na publicação de um livro e diversos textos, além da orientação de alunos em estudos sobre o tema. Também contribuiu para a constituição de um acervo de depoimentos orais e para a orientação da dissertação de Daiana Crús Chagas sobre a erradicação da varíola.^1^

A trajetória foi longa, com muitos alunos, textos e publicações. Trabalhar com esse tema foi extremamente enriquecedor e gratificante. O assunto vacina continua a me instigar. Desde o início, ainda na Ensp, com a pesquisa sobre câncer, a perspectiva de trabalhar com depoimentos orais e as reflexões sobre memória e história me atraíram e me envolveram.

*Na década de 1990, além de atuar nos encontros da Associação Nacional de História (Anpuh), os pesquisadores da Casa promoviam eventos com participação de historiadores que investigavam, nas universidades, história da saúde. Como era esse diálogo?*

Ao longo desse período de minha integração profissional, várias instituições de divulgação foram criadas ou aumentadas, e muitos encontros e congressos foram organizados. A COC e meus parceiros participaram ativamente da organização de eventos que ampliaram nossos diálogos, tanto dentro quanto fora da instituição, favorecendo a consolidação do campo de pesquisa no qual a COC se especializou e que representa de forma singular.

Além da história da vacina e de outros temas, dediquei-me à metodologia de história oral, participando da Associação Brasileira de História Oral ao longo de toda a minha trajetória profissional. Nessa instituição, atuei na diretoria e na promoção de espaços para reflexão e trocas acadêmicas.

*Depois de publicações que se tornaram referência sobre história da varíola, em especial o livro Vacina antivariólica: ciência técnica e o poder dos homens (1808-1920) e alguns artigos sobre o assunto, você se dedicou a outras temáticas e retornou ao estudo da varíola, anos depois, abordando a erradicação dessa doença no século XX e a questão da educação sanitária.*

A publicação, em 1999, do livro *Vacina antivariólica: ciência, técnica e o poder dos homens (1808-1920)*, fruto de minha dissertação de mestrado, que chegou à segunda edição (Fernandes, 2010), foi, para mim, o ponto mais marcante de minha trajetória na história da vacina, configurando-se como o início de minha trajetória como historiadora na COC. Vários aspectos podem ser ressaltados acerca da vacinação antivariólica, como: a relação entre o século XIX (quando a vacina antivariólica foi elaborada) e o início do século XX (quando a vacina passou por processamento e purificação), a erradicação da doença (1970), a ampliação dos processos de imunização para outras doenças e, mais recentemente, na década de 2020, o negacionismo em relação à pandemia de covid-19.

Após me dedicar a outros temas sobre conhecimento científico, doenças e configuração de favelas no Rio de Janeiro, em particular em Manguinhos, retomei a varíola para estudar a erradicação da doença e o papel da educação sanitária para seu controle.

*E você poderia falar um pouco mais sobre esses outros temas?*

O estudo sobre a comunidade científica que atuava em pesquisa sobre plantas medicinais, induzido pela presidência da Fiocruz, favoreceu a elaboração de questionamentos e a produção de minha tese de doutoramento na Universidade de São Paulo, além da produção de um acervo de depoimentos orais sobre o tema. Esse trabalho resultou no livro *Plantas medicinais: memória da ciência no Brasil* (Fernandes, 2004), lançado pela Editora Fiocruz, e em alguns artigos publicados.

Os estudos sobre espaço urbano e favelas, fruto de parceria com o Laboratório Territorial de Manguinhos (da Ensp) solicitada pela direção da COC, levaram à elaboração de duas publicações. O livro *História de pessoas e lugares: memórias das comunidades de Manguinhos* (Costa, Fernandes, 2009), realizado em parceria com Renato Gama-Rosa,^2^ foi publicado pela Editora Fiocruz. O outro livro, *Cidades saudáveis? Alguns olhares sobre o tema* (Silveira, Fernandes, Pellegrini, 2014),^3^ é uma coletânea organizada em parceria com colegas, que inclui um texto escrito por mim junto a outros pesquisadores. Também foi editado pela Fiocruz e laureado com o Prêmio Abeu.

Ainda sobre o tema urbanismo e favelas, realizei um pós-doutoramento na Faculdade de Arquitetura e Urbanismo da Universidade Federal do Rio de Janeiro, buscando diálogo entre história e arquitetura. Fui instigada a questionar a implantação do Programa de Aceleração do Crescimento (PAC – Manguinhos) e seu impacto na vida de alguns moradores, com os quais realizei entrevistas que me levaram a construir textos analíticos, em parceria com André Lima,^4^ meu orientando no mestrado e doutorado. Sobre o PAC, destaco o texto, com inclusão no livro *Pensando as favelas cariocas: história e questões urbanas* (Fernandes, Lima, 2021), organizado por Rafael Gonçalves, Mario Brum e Mauro Amoroso, publicado em 2021.^5^

Ainda narrando minhas diferentes inserções, além da varíola, incluo o período em que desenvolvi pesquisas sobre a história da vigilância sanitária como pesquisadora visitante cedida pela Fiocruz, durante 2006 e 2007, na Universidade Federal da Bahia, no Instituto de Saúde Coletiva. Nesse período, publiquei textos e participei de eventos científicos.

Outro tema que despertou minha curiosidade e no qual investi com uma breve pesquisa surgiu a partir da localização de documentos e de uma entrevista sobre a trajetória de Farmanguinhos (Fiocruz) e do Instituto de Malariologia (RJ). Destaco a relação institucional para a produção de pesticidas destinados ao controle de doenças, questão criticada atualmente, na qual a Fiocruz teve papel importante. Essa reflexão gerou um texto, publicado no volume 13 da coletânea *Uma história brasileira das doenças*, com o título “Doenças endêmicas e a produção de inseticidas no Brasil: do Instituto de Malariologia a Farmanguinhos (1946-1976)” (Fernandes, 2024).

*Como sua pesquisa conecta os desafios históricos e contemporâneos da vacinação, especialmente no que diz respeito à aceitação da vacina, à disseminação do conhecimento científico e às políticas públicas de saúde?*

Em uma pesquisa mais recente, busquei aproximar varíola e vacina da experiência com a epidemia de covid-19, relacionando produção de vacinas, conhecimento científico e respostas sociais quanto às políticas públicas que envolviam essas questões e as duas doenças em seus tempos distintos. Nesse contexto, foi significativa a atuação da mídia na divulgação de conhecimentos científicos, para a qual historiadores, cientistas sociais e profissionais de saúde foram acionados a fim de compartilhar suas reflexões. A vacina antivariólica e a epidemia de gripe espanhola, no início do século XX, funcionaram como referências para a atualização de temas e configuração de novas reflexões. Como um dos resultados, publiquei, em parceria com André Lima (Fernandes, Lima, 2020), um texto na coletânea *Diário da pandemia: o olhar dos historiadores* (Sá et al., 2020).

A recente epidemia de covid-19 também me levou a refletir sobre os temas varíola e vacinação, incluindo o negacionismo que surgiu no Brasil nessa época e a diferença entre esse processo e a negação à vacina verificada ao longo do século XIX e início do XX. Ou seja, a varíola é, para mim, sempre uma questão que me permite atualizar reflexões conforme elas surgem.

Ao longo do século XIX, a vacina contra a varíola gerava muitas dúvidas, pois ainda era um processo bastante incipiente de intervenção em uma doença, o que resultava em resistência tanto no âmbito social quanto no meio médico (Pôrto, Ponte, 2003). Tratava-se da inoculação de doenças similares – varíola e *cowpox* – na expectativa de produzir proteção contra a varíola. Essas práticas, contudo, eram cercadas por dúvidas quanto à possível contaminação do líquido injetado, que poderia provocar outras doenças, ou mesmo a incorporação de características de animais.

Os conhecimentos em microbiologia e imunologia ganharam espaço a partir de meados do século XIX, produzindo efeito, principalmente, quando a imunização pelo *cowpox* foi traduzida em conhecimento científico e levada para o laboratório. No Brasil, essa prática chegou em 1887 e, paulatinamente, foi ganhando adesão ao longo do século XX, refletindo o movimento mundial de incorporação da vacina, apesar das diferenças temporais.

*A Revolta da Vacina, em 1904, é um marco na história do Brasil, revelando tensões entre o Estado e a população no que diz respeito às políticas de saúde pública. A partir de suas pesquisas sobre a história da varíola e da vacinação, como a senhora avalia as ações da população nesse processo?*

A Revolta da Vacina, em 1904, no Rio de Janeiro, foi um marco na história do Brasil e, em particular, na cidade, alterando momentaneamente o rumo da incorporação da vacina como meio profilático contra a doença. Na realidade, esse movimento envolveu não apenas tensões entre o Estado e a população no que diz respeito às políticas de saúde pública e higiene, mas também evidenciou um ambiente de insatisfação social que favoreceu a eclosão da revolta. Esta, além da vacinação, ficou conhecida como Revolta dos Lampiões e funcionou como um estopim da resposta popular. A lei da vacinação obrigatória, com proposta de atualização em 1904, não foi revogada, mas se manteve sem exigência até 1908, quando uma nova epidemia da doença levou à divulgação de seus benefícios e à sua aceitação gradual pela população, juntamente com a vacinação para outras doenças.

No Brasil, a vacinação antivariólica, assim como a imunização contra outras enfermidades, manteve-se obrigatória e foi acatada pela sociedade sem questionamentos significativos ao longo do século XX.

*Quais foram os desafios enfrentados pelo governo ao tentar implementar campanhas de vacinação e quais reflexões podemos elaborar a respeito da relação entre saúde pública, comunicação e adesão popular às campanhas de imunização?*

Durante mais de um século, o país construiu um caminho muito vigoroso de aceitação das vacinas, no qual a divulgação científica e a institucionalização dos conhecimentos e das políticas públicas foram fundamentais. Apesar da inexistência de movimentos de negação e suspeição das vacinas ao longo do século, na década de 1960, o Brasil se colocava como um dos poucos países onde a varíola ainda persistia. A atuação da Organização Mundial de Saúde e da Organização Pan-americana de Saúde, bem como as formulações construídas por profissionais brasileiros para o processo de erradicação, foi fundamental na implementação de uma campanha ativa de vacinação no país, que acabou se tornando referência mundial para a erradicação da doença. De fato, a campanha para a erradicação, fortalecida em meados da década de 1960, traduzia os princípios políticos da época, nos quais a obrigatoriedade se impunha com a vacinação em massa em grandes comícios de apoio ao governo (Hochman, Palmer, 2010).

*Quanto à resistência à vacinação antivariólica ao longo do século XIX no Rio de Janeiro, quais elementos culturais e sociais a senhora poderia elencar para o entendimento desse processo?*

Ao longo do século XIX, podem-se verificar diferentes expressões de resistência à vacinação em todo o mundo. Os processos de produção de vacinas, desde a humanizada até a vacina animal, requereram respostas diferenciadas, tanto populares como dos meios médico e político, com os questionamentos aportados na carência de conhecimento científico sobre os processos de imunização e a microbiologia, que só foram mais bem estruturados após meados do século, em todo o mundo. A produção das vacinas, vinculada ao corpo de um animal, criava incerteza na população e no próprio meio médico quanto à transmissão de doenças próprias dos animais, no caso, os bovinos, ou mesmo de humanos na vacina humanizada.

No Brasil, a institucionalização de medidas preventivas de saúde que incorporassem os saberes produzidos, principalmente nos países europeus, concretizou-se no final do século XIX e início do século XX, com a criação de instituições produtoras de vacina, o aprofundamento de conhecimentos em torno do processo imunológico e da microbiologia, além da implementação de políticas públicas próprias. A legislação de cunho coercitivo praticada no início do século XX produziu resistência popular quanto às medidas de saúde pública, com base no saneamento e na higiene, carentes de medidas educativas, o que acabou levando à aceitação popular pela força e coerção. Ao longo do século, essas medidas foram incorporadas ao cotidiano e, no que diz respeito à vacinação, foram acatadas, pois estavam vinculadas a todas as formas de direitos sociais, como matrículas em escola, casamentos e viagens.

*De que forma podemos diferenciar os conceitos de “negação” à ciência no século XIX e o “negacionismo” político e social observado durante a pandemia de covid-19?*

A negação à vacina que marcou o século XIX e o negacionismo presente diante da epidemia de covid-19 se caracterizam como processos bastante diferenciados, tanto no que tange aos conhecimentos científicos quanto aos aspectos políticos que os sustentam. Negação e negacionismo são bastante distintos nos processos e na abordagem acadêmica. A negação à ciência está calcada no desconhecimento científico, na falta de informação, em interesses políticos, médicos e religiosos, e contra as medidas coercitivas da política estatal. O negacionismo traduz-se como uma terminologia nascida na década de 1940, que trouxe clareza às possíveis leituras quanto a esses fatos, introduzindo uma discussão sobre mentira e veracidade na história, na qual o negacionismo é apontado como um elemento estruturante de poder e superioridade, construído como uma política de Estado com sustentação na desinformação e indução intencional de ignorância científica e histórica.

A experiência brasileira diante da pandemia de covid-19, em uma conjuntura de extremo negacionismo e retrocesso político, científico e social, expressou-se em várias manifestações. O próprio presidente da República^6^ chamou a população ao negacionismo contra a vacinação, que, para ele, produziria mudanças genéticas e de caráter nos vacinados, indicando medicamentos sem comprovação científica e rejeitando o uso de máscaras para a proteção individual e coletiva.

Observaram-se manifestações de ressignificação da Revolta da Vacina de 1904, com construção de eventos de rejeição à vacina, queima de máscaras em praça pública e representações teatrais antivacinas. Após dois anos de pandemia, desestruturação do Sistema Único de Saúde (SUS) e de implantação de dúvidas quanto à veracidade das vacinas e desconfiança nas instituições públicas de saúde, observou-se uma profunda queda na busca pelas vacinas e retorno de algumas doenças antes controladas no país. A covid-19 foi controlada em todo o mundo, com alguns casos e manutenção de vacinação com indicação periódica.

*Para os estudantes e pesquisadores que estão começando suas carreiras nas áreas de história da saúde e das ciências, que conselhos a senhora daria, especialmente em relação à pesquisa interdisciplinar?*

Creio que a multidisciplinaridade e a interconexão entre diferentes disciplinas têm se mostrado muito profícuas para a elaboração de argumentos e a construção de projetos, podendo garantir diversidade de questões que conduzem pesquisas para caminhos inovadores e desafiadores. Meu conselho para os mais novos é que se permitam amplos desafios e que aprofundem conhecimentos que apontem novos caminhos para o bem-estar de nossas sociedades. Ressalto, ainda, a importância da ampliação das estruturas narrativas com leituras para fora da bibliografia específica, mergulhando na literatura romanceada, que traz possibilidades de apreciações diferenciadas das organizações sociais, favorecendo a elaboração de questões e a construção de textos mais criativos.

*Quais as principais fontes e seus respectivos arquivos utilizados para a sua investigação sobre história da vacina antivariólica e educação sanitária?*

A diversidade de fontes a utilizar nas investigações históricas favorece, sem dúvida, a construção de questões ou problemas para as pesquisas. Há uma diferença muito significativa entre o acesso a arquivos e documentos com os quais me deparei na década de 1980, quando iniciei minha carreira, e os momentos atuais, com a internet e o uso de computadores e outros aparatos tecnológicos. As diferentes abordagens, no entanto, mantêm a importância da busca por documentos de valor histórico, impondo, certamente, um volume documental ampliado. Em minhas pesquisas, as buscas manuais em arquivos, incluídos os da própria Fiocruz, me conduziram a ofícios, memorandos, recortes de jornais, legislações e discussões nos espaços legislativos e acadêmicos, além de textos acadêmicos e científicos de época. As charges e as cartilhas institucionais, produzidas no início do século XX, conferiram conteúdos importantes para a perspectiva de um olhar sobre a tradução científica para o popular. Os depoimentos orais, com entrevistas de diferentes personagens que atuaram, principalmente, no processo de erradicação da varíola no Brasil, foram extremamente valiosos para a análise desse momento e a atuação de profissionais brasileiros em outros países.

Em todas as minhas pesquisas com objetos contemporâneos, incorporei entrevistas com os personagens envolvidos, tanto cientistas como populares, constituindo acervos de depoimentos que se encontram sob a guarda da COC.

*Há algum tempo, temos acompanhado iniciativas e reflexões acerca da história pública, o que se acelerou no período da pandemia de covid-19 com historiadores expondo as suas pesquisas para um público mais amplo. Esse processo foi bastante intenso para os historiadores da saúde. Para você, qual a importância desse diálogo entre historiadores da saúde e a sociedade e como ele pode ser feito?*

A experiência com a epidemia mundial de covid-19, que chegou ao Brasil em 2020, abalou a estrutura institucional e a credibilidade social da ciência, quando as vacinas foram rechaçadas e negadas por campanhas negacionistas implementadas pelo próprio governo brasileiro na época. Esse evento proporcionou a pesquisadores de várias áreas estabelecer reflexões e traçar conexões com outros estudos, nos quais a COC teve papel importante, principalmente na perspectiva de aproximação das pesquisas com o público mais amplo. Com a publicação de textos de leitura facilitada, o papel da grande mídia na divulgação dos conhecimentos gerados tem se mostrado profícuo na ampliação do diálogo entre profissionais e sociedade.

## Data Availability

Não estão em repositório.

## NOTAS

1 Daiana Crús Chagas é mestre pelo Programa de Pós-graduação em História das Ciências e da Saúde, com a dissertação *Erradicando doenças: de projeto internacional ao Sistema de Vigilância Epidemiológica: a erradicação da varíola no Brasil (1900-1970)*, em 2008, sob orientação de Tania Fernandes.

2 Renato da Gama-Rosa Costa é professor do curso de Mestrado Profissional em Preservação e Gestão do Patrimônio Cultural das Ciências e da Saúde, da Fiocruz.

3 Livro organizado em parceria com Carmen Beatriz Silveira, doutora em planejamento urbano e regional pelo Instituto de Pesquisa e Planejamento Urbano e Regional da UFRJ, e Bárbara Pellegrini, mestre em comunicação e cultura pela Escola de Comunicação da UFRJ.

4 André Luiz da Silva Lima é mestre e doutor pelo Programa de Pós-graduação em História das Ciências e da Saúde e atua na Coordenação Colegiada do Projeto de Promoção de Territórios Saudáveis e Sustentáveis da Cooperação Social da Presidência da Fiocruz.

5 Rafael Soares Gonçalves é doutor em história e civilizações pela Université de Paris VII – Denis Diderot e professor do Departamento de Serviço Social da Pontifícia Universidade Católica do Rio de Janeiro. Mario Brum é doutor em história pela Universidade Federal Fluminense e professor no Departamento de História da Universidade do Estado do Rio de Janeiro. Mauro Amoroso é doutor em história, política e bens culturais pela Fundação Getulio Vargas e professor da Faculdade de Educação da Baixada Fluminense da Universidade do Estado do Rio de Janeiro.

6 Na época, o presidente era Jair Bolsonaro.
